# Incorporating abundance information and guiding variable selection for climate-based ensemble forecasting of species' distributional shifts

**DOI:** 10.1371/journal.pone.0184316

**Published:** 2017-09-08

**Authors:** Evan P. Tanner, Monica Papeş, R. Dwayne Elmore, Samuel D. Fuhlendorf, Craig A. Davis

**Affiliations:** 1 Department of Natural Resource Ecology and Management, Oklahoma State University, Stillwater, Oklahoma, United States of America; 2 Department of Ecology and Evolutionary Biology, University of Tennessee, Knoxville, Tennessee, United States of America; University of Sydney, AUSTRALIA

## Abstract

Ecological niche models (ENMs) have increasingly been used to estimate the potential effects of climate change on species’ distributions worldwide. Recently, predictions of species abundance have also been obtained with such models, though knowledge about the climatic variables affecting species abundance is often lacking. To address this, we used a well-studied guild (temperate North American quail) and the Maxent modeling algorithm to compare model performance of three variable selection approaches: correlation/variable contribution (CVC), biological (i.e., variables known to affect species abundance), and random. We then applied the best approach to forecast potential distributions, under future climatic conditions, and analyze future potential distributions in light of available abundance data and presence-only occurrence data. To estimate species’ distributional shifts we generated ensemble forecasts using four global circulation models, four representative concentration pathways, and two time periods (2050 and 2070). Furthermore, we present distributional shifts where 75%, 90%, and 100% of our ensemble models agreed. The CVC variable selection approach outperformed our biological approach for four of the six species. Model projections indicated species-specific effects of climate change on future distributions of temperate North American quail. The Gambel’s quail (*Callipepla gambelii*) was the only species predicted to gain area in climatic suitability across all three scenarios of ensemble model agreement. Conversely, the scaled quail (*Callipepla squamata*) was the only species predicted to lose area in climatic suitability across all three scenarios of ensemble model agreement. Our models projected future loss of areas for the northern bobwhite (*Colinus virginianus*) and scaled quail in portions of their distributions which are currently areas of high abundance. Climatic variables that influence local abundance may not always scale up to influence species’ distributions. Special attention should be given to selecting variables for ENMs, and tests of model performance should be used to validate the choice of variables.

## Introduction

Global climate change may markedly influence species populations worldwide and may have increased negative effects on species that are not able to adapt to changes in climate or to disperse to suitable conditions elsewhere [1–3]. Climatic conditions are important in determining an organism’s geographic distribution because of specific eco-physiological constraints [2, 4–6]. Climate change has already caused shifts in the distribution of many species [7–9], and is estimated to continue affecting distributions in the future [10, 11]. Ecological niche models (ENMs) can be useful in predicting changes in a species’ distribution [12], though such techniques often rely on the availability of a sufficient amount of occurrence and/or abundance data representative of the species’ distribution [13]. This potential limitation has led to a large number of studies focused on avian species [14,15] because of the plethora of occurrence data publicly accessible through government monitoring programs (Breeding Bird Survey [BBS]; [16]), as well as citizen science programs (eBird; [17]).

Though initially ENMs were focused on studying the biogeography of species, more recently research has focused on utilizing such models to help relate probability of occurrence to intrinsic growth rates [18], population size [19], population density [20], reproductive parameters [21], and species abundance [22–24]. Combined with population demographic information, these analyses can be used to more accurately target areas of conservation concern [25] by identifying potential “species’ strongholds”. However, an important assumption made about climatic variables incorporated in ENMs is that they are biologically meaningful to the species of interest, and the selection of these variables can greatly affect the performance and resulting ENMs [26–27]. When incorporating population demographic information to ENMs, inclusion of biologically meaningful variables that directly relate to demographic rates and exclusion of “relaxed” variables [27], or variables that have little importance on such rates, should be taken into consideration [28]. Despite this concern, ecological niche modeling studies often use all 19 bioclimatic variables that are freely available (www.worldclim.org) and correlation/variable contribution filtering to dictate the modeling parameters [29]. Such generalized approaches may cause major sources of uncertainty when creating ENMs [30].

Here, we investigate model performance differences under three variable selection methods and present an analysis of potential climate induced shifts in the distributions of the temperate North American quail species (California quail [*Callipepla californica*], Gambel’s quail [*Callipepla gambelii*], scaled quail [*Callipepla squamata*], northern bobwhite [*Colinus virginianus*], Montezuma quail [*Cyrtonyx montezumae*], and mountain quail [*Oreortyx pictus*]). The three species in the *Callipepla* genus are primarily distributed within the western and southwestern regions of North America. More specifically, California quail are distributed from parts of southern Washington, through Oregon and California, and into Baja California and generally inhabit semiarid regions characterized by vegetation ranging from open woodlands, shrublands, and agricultural areas [31]. Gambel’s quail are distributed in the arid southwestern regions of North America and inhabit areas indicative of desert vegetation but are also abundant within agricultural and urban areas [32]. Scaled quail are distributed throughout the arid and semi-arid southwestern and south-central regions of North America and primarily inhabit areas with grassland and shrubland vegetation [33], though will often be present within agricultural areas as well [33–34]. Similarly, the mountain quail and Montezuma quail are also western species in North America, but mountain quail typically inhabit upland forests generally above 1,000 m in elevation [35]. Conversely, the Montezuma quail are distributed in the arid southwestern portions of the United States and throughout Mexico and inhabit woodlands typically comprised of oak (*Quercus* spp.) and oak-pine (*Pinus* spp.) species [36]. Finally, the northern bobwhite is the only species that is primarily found in the eastern regions of North America, where its distribution includes the southeastern United States, though ranges north between 40–45° latitude, west into the Great Plains, and into parts of northern Mexico [37–38]. The northern bobwhite is a shrubland obligate species, but does also occur in grasslands, woodland savannahs, and small agricultural areas throughout its distribution [38–39].

These species are of conservation concern as they have experienced distribution-wide declines in recent decades [40–41], which may be exacerbated in future climates because of their low dispersal abilities [42]. Furthermore, many of these species are designated as umbrella species [43] for biodiversity conservation and have been shown to be positive indicators for the occurrence of other avian species of conservation concern [44]. Thus understanding potential climate induced shifts in the distributions of these species may have conservation implications beyond temperate North American quail. Finally, these species offer an opportunity to relate abundance data to distributional estimates obtained from ENMs because extensive knowledge exists on what abiotic variables influence local annual abundance and reproduction of most of these species (Table 1). Therefore, our objectives were to: 1) test whether performance of ENMs improved when using only variables known to directly affect species’ abundance, compared to performance of models based on other variable selection approaches and 2) use the top performing ENMs from our variable selection exercise to analyze species abundance data in relation to future distribution shift estimates to identify potential critical areas of loss in environmental suitability. We hypothesized that there would be idiosyncratic relationships between the best variable selection approaches across species, which may be driven by scale mismatch between what influences local abundance and what influences broad scale distributions. Furthermore, as we generally expect future distributions of our target species to shift in relation to changing climatic conditions [7–9], we also hypothesized that species with the highest areas of abundance located along the fringe of their distributions (i.e., northern bobwhite [41]) would lose potential population “strongholds” (i.e., areas of high relative abundance) due to changes in future distributions based on climate change projections.

**Table 1:** Climate variables known to affect abundance and/or reproduction of temperate quail species in North America.

Common name	Scientific name	Climate variable	Reference(s)
California quail	*Callipepla californica*	Fall-Spring precipitation	[45]
		Winter precipitation	[46]
Gambel's quail	*Callipepla gambelii*	Winter-Spring precipitation	[47–50]
		Maximum July temperature	[50]
Scaled quail	*Callipepla squamata*	Winter precipitation	[51]
		Summer precipitation	[52–54]
		Spring precipitation	[52–53]
		Annual precipitation	[55]
		Modified Palmer Drought Severity Index^a^	[55]
Northern bobwhite	*Colinus virginianus*	Maximum July temperature	[56–57]
		Spring precipitation	[58]
		Summer precipitation	[58]
		Fall precipitation	[57]
		Modified Palmer Drought Severity Index^a^	[55,59]
		Annual precipitation	[59]
Montezuma quail	*Cyrtonyx montezumae*	Summer precipitation	[60]
Mountain quail	*Oreortyx pictus*	N/A^b^	N/A^b^

^a^ Not included in our analysis.

^b^ Information not available.

## Materials and methods

To test our hypotheses, we used a presence-only maximum entropy algorithm to build ENMs with variables known to directly influence species abundance at a local level. We then compared the performance of these models to ENMs built using traditional variable selection approaches to determine if factors influencing local abundance data also scaled to influence environmental suitability across species’ distributions. Finally, we forecasted species distributional shifts under future climate scenarios through the use of our top performing ENM to determine if areas of currently high relative species abundance along the edge of distributions would be lost under the future climate scenarios.

### Species occurrence data

We collated species occurrence data from the BBS [16] and eBird [17] databases, similar to other ENMs studies [15,61–62], to create a presence-only occurrence dataset for our modeling exercises. The BBS is a multi-national bird survey program that has been used to monitor breeding bird population trends in North America since 1966 [63–64]. Its design includes using thousands of observers annually to conduct point count surveys along repeated transects located on roadways throughout much of North America [63]. Raw data and trend estimates are made publicly available through the BBS website (https://www.pwrc.usgs.gov/bbs/). A more detailed description of the BBS protocol and analysis techniques are provided by [63] and [65]. The eBird database is a citizen science program established to archive and share bird observations submitted by the public [17]. Currently, this is considered the largest ecology based citizen science project [61]. Inclusion of eBird records in our occurrence dataset allowed us to consider geographic areas outside the sampling range of the BBS survey (i.e., Mexico) in which some of our target species occur. The range of dates for occurrence data from the BBS and eBird was 1966–2000 and 1950–2000, respectively, which temporally matched the range in dates for the climatic variables included in the modeling framework discussed below. We note that eBird observations were more abundant in recent decades as opposed to the earlier decades of our study timeframe, however this database was useful in obtaining occurrence information through the entire temporal range that coincided with our climatic data.

Because the species we examined are non-migratory game species, the overall number of occurrence points was much greater than typical sample sizes recommended for ENMs [66]. Oversampling and clustering of occurrence data can often lead to overfitting issues in a presence-only modeling framework [67–68]. This relates to models fitting tightly to calibration data, which in turn will limit the ability of the model to predict independent evaluation data [68]. Spatially rarefying occurrence data in such situations has been shown to improve models by limiting the possibility of over-fitting [68–69]. Previous studies vary in the spatial rarefication buffer used (10-20km), with justification for these buffer distances based on ecology of the study species [69], spatial heterogeneity of the climate [68], or the clustering nature and abundance of data points from a database [15]. Similar to [15], we chose a 20 km buffer around points for all six species we examined, a distance within their dispersal range [70–73]. To spatially rarefy occurrence data, we used the Spatially Rarefy Occurrence Data tool in the SDM Toolbox (v1.1b; [74]) for ArcGIS 10.2.1 (ESRI, Redland, California, USA). Further elimination of points included the removal of occurrence points that represented “introduced” or “stocked” populations, as we were only interested in modeling the distribution of native populations. To eliminate these types of entries, we removed any occurrences outside the known historic distribution of the species and any entries in which observers noted “stocked” or “introduced” individuals in the comments section. We also verified that locations were within the species’ historic distribution by validating our data with range maps downloaded from NatureServe [75]. This resulted in the removal of five and 222 occurrence locations for Gambel’s quail and California quail, respectively. No other species had occurrence locations removed after data validation with NatureServe maps. Finally, as outlined by [15], eBird includes different observation protocols that may influence the interpretation of occurrence type. The “exhaustive area counts” protocol can represent single occurrence coordinates for large areas covered by the observer and may not reflect occurrence at a scale relevant to ecological modeling. Likewise, the “traveling count” protocol represents a single occurrence coordinate for a large distance traveled. To account for these potential biases, we eliminated traveling count observations in which the observer traveled >2 km [15,76] and exhaustive area counts in which the observer covered an area >100 ha [15].

Initial sample sizes and spatially rarefied sample sizes varied across species. Initial sample sizes ranged from 382 (Montezuma quail) to >38,000 (bobwhite) occurrence locations. After spatially rarifying our data, sample sizes were reduced to: 552, 268, 317, 2,013, 31, and 216 for California quail, Gambel’s quail, scaled quail, northern bobwhite, Montezuma quail, and mountain quail, respectively (S1 Fig).

### Climate data

We obtained baseline (1950–2000) climate data at a spatial resolution of five arc minutes (~9 km) from the WorldClim database [77], which represented static climatology raster surfaces (i.e., a mean value from 1950–2000) and included 19 bioclimatic variables as described by [77]. Additionally, we derived five average maximum summer temperature and cumulative seasonal rainfall variables from WorldClim monthly average climate data: average maximum temperature for June, July, and August (°C), cumulative rainfall for winter (mm; December, January, and February), cumulative rainfall for spring (mm; March, April, and May), cumulative rainfall for summer (mm; June, July, and August), and cumulative rainfall for fall (mm; September, October, November). These five variables, along with three of the 19 bioclimatic variables, maximum temperature of the warmest month (°C; Bio5), mean annual temperature (°C; Bio1), and average annual rainfall (mm; Bio12), represented variables that have been demonstrated to directly influence the abundance of our study species based on previous research (Table 1).

Previous research has emphasized the importance of training ENMs only based on climatic data existing within the known spatial distribution of a study species [78–79]. We therefore trained our ENMs with climatic data that were clipped to the spatial extent of the species’ potential study extent. We restricted the study extent to a 500km buffer around “contemporary” locations [15]. [15] described contemporary locations as species occurrence points from the year 2001. As our most recent occurrence data was in 2000, we considered these locations to be our contemporary points which were used in creating the species’ study extent. In a similar study, a 200km buffer was shown to be too restrictive for many species and their projected future distributions [22], thus [15] suggested a 500km buffer to encompass potentially large shifts in projected occurrence data. Therefore, we used this buffered range as our study extent for selecting background points and projecting future species’ distributions. This buffer ensured that no occurrence points were located outside of our study extent for each species. Furthermore, the species’ specific study extents created from these contemporary locations included the known natural historic ranges of all six species, as corroborated by experts of these species (described in [80–81]). Study extents after this procedure were: 1.80x10^12^ ha, 2.39x10^12^ ha, 2.63x10^12^ ha, 3.29x10^12^ ha, 3.43x10^12^ ha, and 7.43x10^12^ ha for mountain quail, Montezuma quail, Gambel’s quail, scaled quail, California quail, and northern bobwhite, respectively. We projected all of our data into the North American Albers Equal Area Conic projection [67,82] as our study extents covered a large range in latitude (>200 km) based on our criteria [67].

### Maximum entropy modeling

#### Preliminary modeling for variable selection

We created seven unique suites of climatic variables to run seven separate model suites, thus we directly tested whether or not a model using climatic variables known to directly affect local abundance performed better than other approaches. The seven model suites used were: biological (use of variables known to directly affect local abundance [Table 1]), correlation/variable contribution (CVC; i.e., variable reduction through correlation analysis [29] and variable contribution to model accuracy gain), and random (i.e., a selection of random bioclimatic variables equal to the number of variables contained in each biological model [*n* = 5 random suites]).

We used the maximum entropy algorithm Maxent, version 3.3.3k [83] to test which variable selection approach performed best for creating ENMs. The Maxent algorithm is used for generating ENMs with presence-only/pseudo-absence data [84] and climatic variables. Maxent has been shown to have higher predictive power than many other modeling techniques [66–67] by minimizing the entropy (a measure of dispersedness) between the probability densities of presence data and “background” data (locations without presence information) in environmental covariate space [67]. For our preliminary Maxent modeling (i.e., for the variable selection exercise), input parameters were kept at default values [83]. Though changing the input parameters from default values can influence model performance [85–87], we did not change input parameters until the second (and final) stage of our modeling efforts (described in the next section), which was done after our initial variable selection analysis was completed. This included the use of 10,000 background points, which has been shown to perform similarly when compared to models using all potential background points [83]. We used a regularization multiplier of 1, performed 500 iterations per model, and used a convergence threshold of 0.00001 for each model. To test the validity of our models, we held-out 25% of our presence data for testing through random selection and used 75% for training each species model [15,88–89]. We replicated models for each variable suite 100 times using the bootstrap method. For each model, we used 10 percentile training presence as the threshold method to convert the continuous occurrence probability estimates into binary suitability maps. This threshold rule has been shown to outperform other threshold rules in Maxent modeling [90]. Thus, any cells with logistic values below these individual threshold values were categorized as unsuitable.

To create the CVC suite, we initially selected and eliminated highly correlated variables from the 19 bioclimatic variables (|*r*|>0.7; [29]), as well as variables contributing ≤1% to model accuracy gain [21] as determined through Maxent. If two variables were highly correlated, we initially eliminated a correlated variable if it contributed ≤1% to model accuracy gain. If both correlated variables contributed >1% to model accuracy gain, we retained the variable contributing most to model accuracy gain and eliminated the second variable. This was repeated until all pairwise correlation coefficients between our climatic variables was |*r*|≤0.7 and all variables contributed >1% to model accuracy gain. For the random suites, we used randomly selected variables from the list of the 19 bioclimatic variables. The number of randomly selected variables was equal to the number of variables used in our biological model. Because the performance of a model built with random variables is highly determinant on the variables being selected, we compared models based on five suites of random variables to the models obtained with CVC and biological suite of variables. For the biological suite, we limited our variable selection to the eight climatic variables that were based on previous knowledge of these species’ ecological responses to environmental trends, as described in the “Climate Data” section (Table 1). Similar to the CVC modeling approach, we estimated a Pearson’s correlation coefficient for all combinations of our biologically relevant variables and used the threshold of |*r*|>0.7 to eliminate highly correlated variables [29]. We also eliminated variables that had ≤1% contribution to accuracy gain of preliminary models that we ran for the six species [21]. Thus, each species of interest had a unique set of variables for their respective biological model suites.

To evaluate and compare the performance of our seven model suites in Maxent, we used test occurrence data and the binary suitability maps to calculate test omission error, averaged across the 100 replicates for each suite and standardized by mean area predicted present [91], as well as the average Area Under the Curve (AUC) of the Receiver Operating Characteristic (ROC), a threshold independent method of evaluating models [92]. Since test omission errors are sensitive to the amount of area predicted suitable [93], we further assessed the performance of our model suites using the standardized omission error. This is calculated by estimating test omission for each model replicate based on a binary suitability map that has the same percent area of suitability, which was set at the mean percentage of suitable area predicted across all model replications for each species [91]. This standardized test omission error thus allows for direct comparison of performance between models. For the threshold-independent method of model evaluation (ROC), the AUC value can range from 0–1 and indicates the probability of a presence point having a higher AUC value than a random background point. This means that a value of 1 indicates a completely accurate prediction, whereas a value of 0.5 indicates no difference in the presence and the background point, and values <0.5 indicate predictions that perform worse than a random model [94]. The AUC value has been scrutinized for being an unreliable predictor of model performance [95–96], thus conclusions based solely on the AUC of the ROC are not recommended. For this reason, we selected the variable suite that had the best performing standardized test omission error and then carried forward that variable suite for further Maxent models described in the next section. We tested for differences in the average standardized test omission errors by conducting a one-way ANOVA and a post hoc Tukey HSD pair wise comparisons test (α = 0.05) across model suites using PROC GLM in SAS 9.4 (Statistical Analysis System Institute Inc, Cary, North Carolina, USA). If there were no statistical differences in the top performing suites, we used the biological suite as our baseline model to maintain a better “ecological understanding” of our study species [29].

#### Accounting for Maxent model complexity

Incorporating species occurrence data and climatic variables from our best performing variable suite described above, we estimated current and future quail distributions with Maxent, version 3.3.3k [83]. As mentioned in the previous section, algorithm parameter values have been shown to influence the performance of models created through Maxent [85–87]. Specifically, the regularization multiplier, which controls model complexity, can significantly influence the performance of models when this parameter is changed from its default value of one [87]. To account for this, we calibrated our best performing models for each species from the variable selection analysis with different values for the regularization multiplier. We compared average test omission rates across models with different regularization multiplier values (0.25, 0.50, 1.00, 1.50, 2.00, 4.00, 6.00, 8.00, and 10.00; [87]) across 100 replicates. Beyond changing the regularization multiplier values, all modeling efforts at this stage of the analysis were the same as described in the previous section. We tested for differences in average test omission errors by conducting a one-way ANOVA and a post hoc Tukey HSD pair wise comparisons test (α = 0.05) across models with differing regularization multiplier values using PROC GLM in SAS 9.4. We used the regularization multiplier that resulted in the statistically lowest test omission error for all further analyses, unless the default regularization multiplier value (1.00) was not statistically different than other regularization multiplier values. Finally, we projected our species’ specific Maxent models onto the future climate change scenarios described below.

### Future projections and post-modeling analysis

To model species distributional shifts under future climate scenarios, we carried forward the best performing model for each species from the two previous stages of model building as our baseline model. Climate data for future projections (the same variables as for baseline models) were also obtained from the WorldClim database at a spatial resolution of five arc minutes (~9 km), similar to the baseline data. To account for variation in global circulation models (GCMs) on which the future climate datasets are based, we used an ensemble forecasting procedure to estimate future distribution shifts [97]. To capture variability across GCMs, we randomly selected four [98] and used data at four representative concentration pathways (RCPs; 2.6, 4.5, 6.0, and 8.5), or scenarios of greenhouse gas emissions, across two time periods (2050 [average for 2041–2060] and 2070 [average for 2061–2080]) in which data were available. The four random GCMs selected were the CCSM4, GISS-E2-R, HadGEM2-ES, and the MRI-CGCM3, all included in the 5^th^ Assessment IPCC report (AR5; [99]). In sum, for each species we estimated 32 baseline models and 32 corresponding future projections (4 GCMs X 4 emission scenarios X 2 time periods).

After estimating ENMs under future climate scenarios, we used the Raster Calculator tool in ArcGIS 10.2 to compare differences in binary occurrence probabilities of current and future distributions. An ensemble suitability range for current distributions was assigned where all 32 model runs agreed on a binary presence for each species. We then created ensemble future distribution projections across both time periods, at three levels of model projection agreement: 75%, 90%, and 100% (i.e., where 75%, 90%, and 100% of the 32 models agreed). We used binary outputs to create our ensemble forecasts as to avoid uncertainty in the appropriateness of averaging different Maxent logistic values across models. We included three levels of agreement to capture variability between climate scenarios that may have altered degree of agreement. Based on these ensemble forecasts, we categorized distribution conditions that raster cells could be classified into 8 conditions (Table 2). We used these distributional conditions to estimate the overall percent gain or loss for future distributions of the six quail species, relative to the current estimated distribution.

**Table 2:** Possible distribution conditions occurring within species’ potential distribution maps produced by the Maxent algorithm under future climate scenarios.

Condition	Description
1	distribution expansion from current to 2050 and remaining suitable from 2050 to 2070
2	suitable at current and through all time periods
3	unsuitable from current to 2050 but expanding from 2050 to 2070
4	distribution contraction from current to 2050 but expanding from 2050 to 2070
5	distribution expansion from current to 2050 but contracting from 2050 to 2070
6	suitable from current to 2050 but contracting from 2050 to 2070
7	unsuitable at current and through all time periods
8	distribution contraction from current to 2050 and remaining unsuitable from 2050 to 2070

Both Montezuma quail and mountain quail can inhabit regions classified as “sky islands” and are often restricted to areas of elevations ≥1,000 m [35, 100]. For these two species, we used the estimated shifts in species distributions based on our 90% model agreement to determine whether or not areas that were predicted to become unsuitable in future climate scenarios were lower in elevation compared to areas predicted to remain suitable with a t-test assuming unequal variance (*p* < 0.01). To do this, we obtained a 30 arc-second (~1 km) digital elevation model (DEM) raster online (https://databasin.org/datasets/d2198be9d2264de19cb93fe6a380b69c) from a collaborative effort between the National Aeronautics and Space Administration, the United Nations Environment Programme/Global Resource Information Database (UNEP/GRID), the U.S. Agency for International Development (USAID), the Instituto Nacional de Estadistica Geografica e Informatica (INEGI) of Mexico, the Geographical Survey Institute (GSI) of Japan, Manaaki Whenua Landcare Research of New Zealand, and the Scientific Committee on Antarctic Research (SCAR).

Finally, we accessed relative abundance data (S2 Fig) for all species except the Montezuma quail (in which data were not available [41]) to determine the implications of future distributional shifts on current populations. For each of the other five species, abundance was estimated from BBS data for 2008–2012. We conducted a two-way ANOVA and a post hoc Tukey HSD pair wise comparisons test (α = 0.05) to test for statistical differences between relative abundance values among areas of current suitability to areas that are estimated to contract (or become unsuitable) in future climate scenarios and across species using PROC GLM in SAS 9.4. Tests were conducted across our distribution conditions (Table 2) and across 75%, 90%, and 100% ensemble forecasts. We initially tested for differences in relative abundance across suitability conditions for our entire dataset with species as a random effect. If species was considered significant in our model, we conducted a one-way ANOVA to estimate species-specific relationships. Because sample sizes were large (*n* >1,000) for our ANOVA tests on the abundance data for all species, we estimated ƞ^2^ (Eta squared) to test for an effect size between the possible distribution conditions [101]. We considered ƞ^2^ < 0.06 to be a small effect size, ƞ^2^ 0.06 to <0.14 to be a medium effect size, and ƞ^2^ ≥0.14 to be a large effect size [101]. Relative abundance data not within the species native historic range was not included in these analyses. Furthermore, as relative abundance data were from 2008–2012, data outside the continental United States were not available.

## Results

### Variable selection and model performance

**Fig 1:**
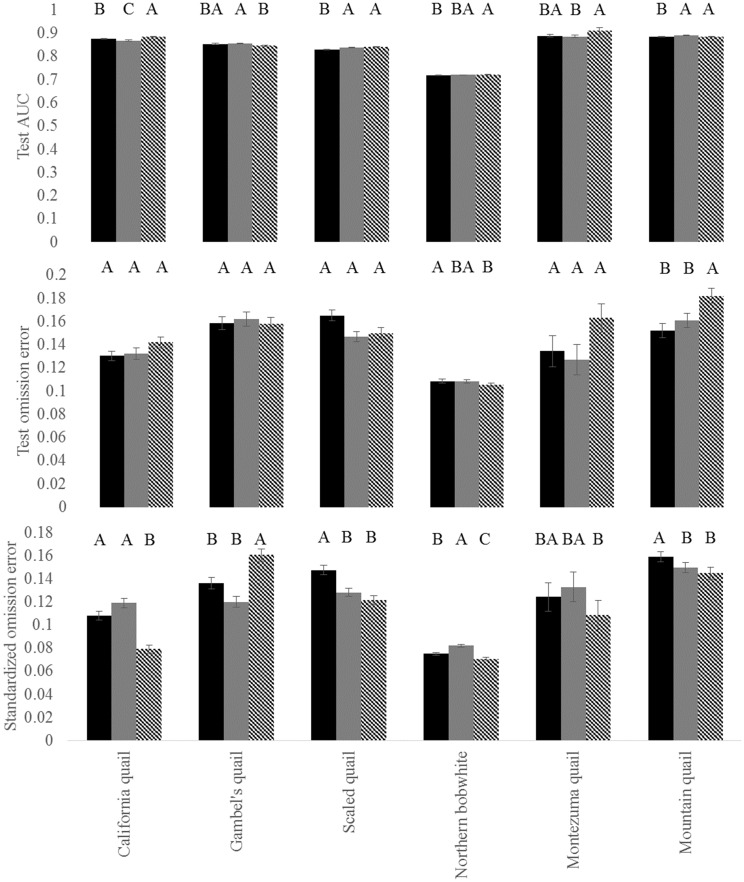
Model performance metrics used in determining the best variable selection approach to estimate potential distributions for temperate North American quail through the Maxent algorithm. Variable selection approaches included a biologically relevant suite (black bar), the top performing random suite (grey bar), and a correlation/variable contribution (striped bar) suite. The random variable suite included in this figure is the random suite that had the best standardized omission error of the five random variable suites included in our analysis. Significant differences in model performance metrics are indicated by letter groupings from post hoc Tukey HSD pairwise comparison test results from a significant one-way ANOVA.

The biological variable suite was only used for two species in our modeling framework, suggesting there was evidence that variables that influence local species abundance do not necessarily scale to influence species’ distributions. More specifically, based on standardized test omission error, the CVC variable selection approach significantly outperformed the biological variable selection approach and random variable selection approach (Fig 1) for the California quail (*F*[6,693] = 83.93, *p* = <0.01) and northern bobwhite (*F*[6,693] = 98.84, *p* = <0.01). Furthermore, the CVC variable selection approach significantly outperformed the biological variable selection approach for the scaled quail (*F*[6,693] = 59.27, *p* = <0.01) and mountain quail (*F*[6,693] = 28.76, *p* = <0.01), though was not statistically different than the top performing random variable suite (*p* >0.05). For Gambel’s quail, the biological variable suite significantly outperformed the CVC approach (*F*[6,693] = 28.11, *p* = <0.01), though was not statistically different than the top performing random variable suite (*p* >0.05). Finally, although the biological, CVC, and top performing random variable suite outperformed the four remaining random variable suites (*F*[6,693] = 4.59, *p* = <0.01), there were no significant differences between the biological, CVC, and top performing random variable suite when analyzing Montezuma quail data (Fig 1). Models for all species and all variable suites besides one random variable suite for bobwhite performed reasonably well [102], with all test AUCs averaging within 0.72 to 0.91 (Fig 1). One random variable suite for bobwhite had a test AUC value of 0.67. Test omission rates at the 10% training omission threshold also indicated that our models performed well, with average rates ranging from 0.11 to 0.18 (Fig 1). Based on the standardized test omission error values, we used the CVC variable suite for California quail, scaled quail, mountain quail, and bobwhite ENMs. We used the biological variable suite to create ENMs for the Gambel’s and Montezuma quail.

**Fig 2:**
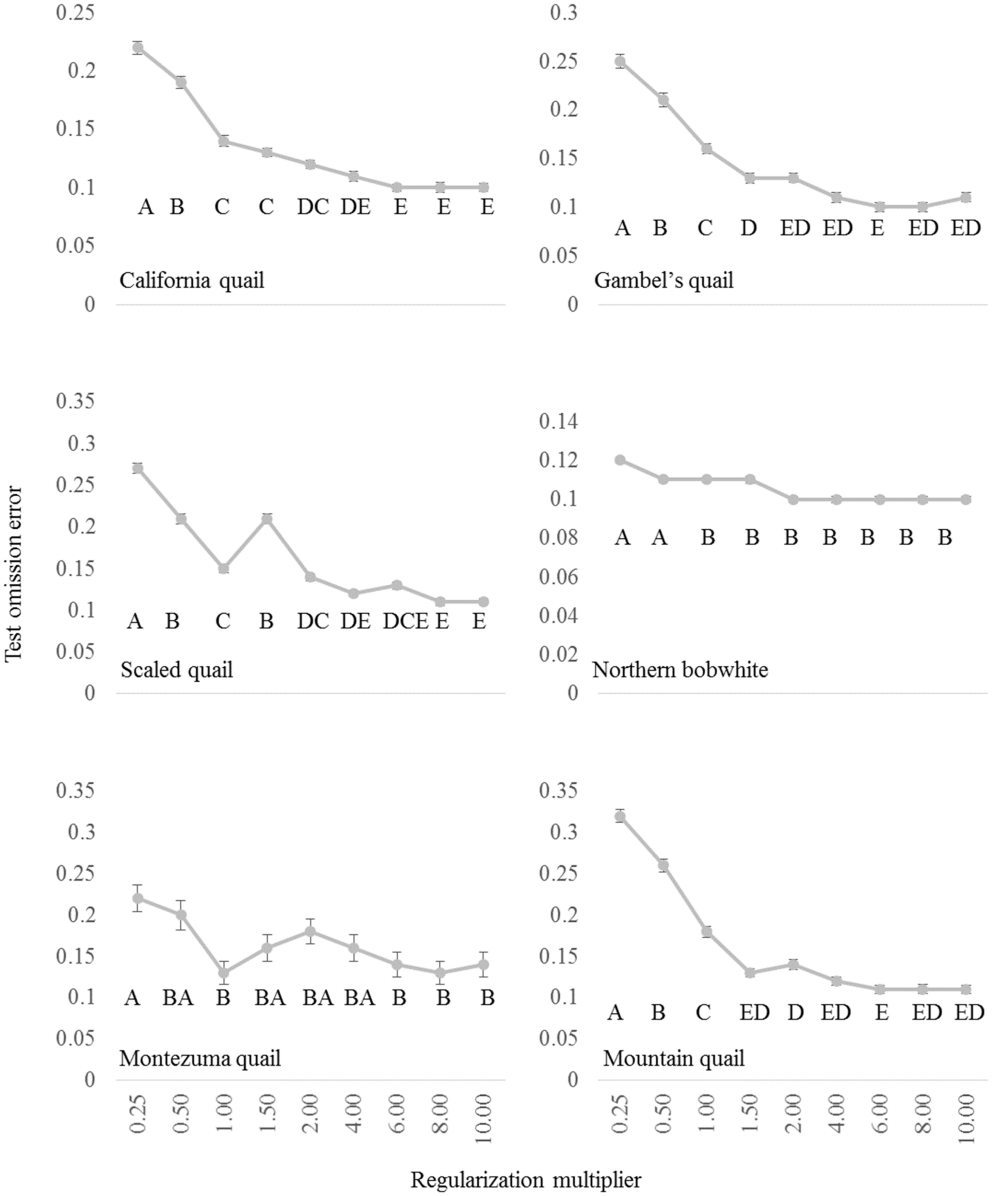
Test omission errors from regularization multiplier tuning experiments of Maxent models for temperate North American quail. Test omission errors were based on a 10^th^ percentile presence threshold and are averaged across 100 replications. Significant differences in test omission estimates are indicated by letter groupings from post hoc Tukey HSD pairwise comparison test results from a significant one-way ANOVA.

Test omission errors for all six species were generally greater for models when the regularization multiplier was below the default value of 1.00 (Fig 2). The one-way ANOVA results indicated that there were statistical differences in test omission errors across the range of values for the regularization multiplier: California quail (*F*[8,891] = 97.58, *p* <0.01), Gambel’s quail (*F*[8,891] = 87.21, *p* <0.01), scaled quail (*F*[8,891] = 130.16, *p* <0.01), northern bobwhite (*F*[8,891] = 17.48, *p* <0.01), Montezuma quail (*F*[8,891] = 3.94, *p* <0.01), and mountain quail (*F*[8,891] = 162.53, *p* <0.01). However, the default regularization multiplier value was used for northern bobwhite (*p* > 0.82) and Montezuma quail (*p* > 0.48) models as there was no statistical difference in model performance between a default value and other values with low test omission values (Fig 2). Conversely, regularization multiplier values of 8.00, 6.00, 6.00, and 8.00 were used for California quail, Gambel’s quail, mountain quail, and scaled quail as these values outperformed models made with other regularization multiplier values based on test omission errors.

Average variable contributions to model accuracy gain (averaged across 100 replicates per species) are indicated in Table 3. The Montezuma quail and the northern bobwhite had the least number of contributing variables whereas scaled quail and mountain quail had the most, after adjusting models for initial variable correlations and contributions. At least one bioclimatic variable was included in each species’ model set except for the Gambel’s quail. Mean temperature of the wettest quarter (BioClim8) was the most frequently included variable and occurred in four of the six species’ ENMs. Average partial plot relationships between all contributing models and climate suitability were idiosyncratic for all species (S3–S8 Figs).

**Table 3:** Variables used^a^ in the final Maxent experiments for training ecological niche models of temperate North American quail species and average variable contribution to model accuracy gain. Standard errors are in parentheses.

Variable	California quail	Gambel's quail	Scaled quail	Northern bobwhite	Montezuma quail	Mountain quail
BioClim^b^ 1	0.00	0.00	0.00	49.62 (0.17)	26.83 (0.74)	0.00
BioClim 3	25.28 (0.49)	0.00	19.54 (0.57)	0.00	0.00	9.78 (0.45)
BioClim 4	0.00	0.00	0.00	0.00	0.00	31.48 (1.00)
BioClim 8	18.40 (0.61)	0.00	7.84 (0.37)	1.55 (0.06)	0.00	12.60 (0.32)
BioClim 9	4.55 (0.19)	0.00	0.00	0.00	0.00	0.00
BioClim 11	0.00	0.00	22.00 (0.64)	0.00	0.00	0.00
BioClim 14	0.00	0.00	0.00	0.00	0.00	11.62 (0.32)
BioClim 15	0.00	0.00	7.67 (0.41)	36.54 (0.16)	0.00	11.73 (0.77)
BioClim 16	0.00	0.00	34.29 (0.81)	0.00	0.00	0.00
BioClim 18	38.93 (0.54)	0.00	0.00	12.29 (0.20)	0.00	0.00
BioClim 19	12.84 (0.58)	0.00	8.66 (0.25)	0.00	0.00	22.79 (0.74)
Cumulative fall precipitation	0.00	2.49 (0.22)	0.00	0.00	0.00	0.00
Cumulative spring precipitation	0.00	19.44 (0.74)	0.00	0.00	25.11 (0.70)	0.00
Cumulative summer precipitation	0.00	18.60 (0.36)	0.00	0.00	30.86 (0.73)	0.00
Cumulative winter precipitation	0.00	5.31 (0.21)	0.00	0.00	17.20 (0.80)	0.00
Maximum average summer temperature	0.00	54.16 (0.77)	0.00	0.00	0.00	0.00

^a^ Variables with 0% contribution to model accuracy gain were not used in model training.

^b^ BioClim variables are estimated from [77] and are described at www.worldclim.org.

### Future species’ distributions

**Fig 3:**
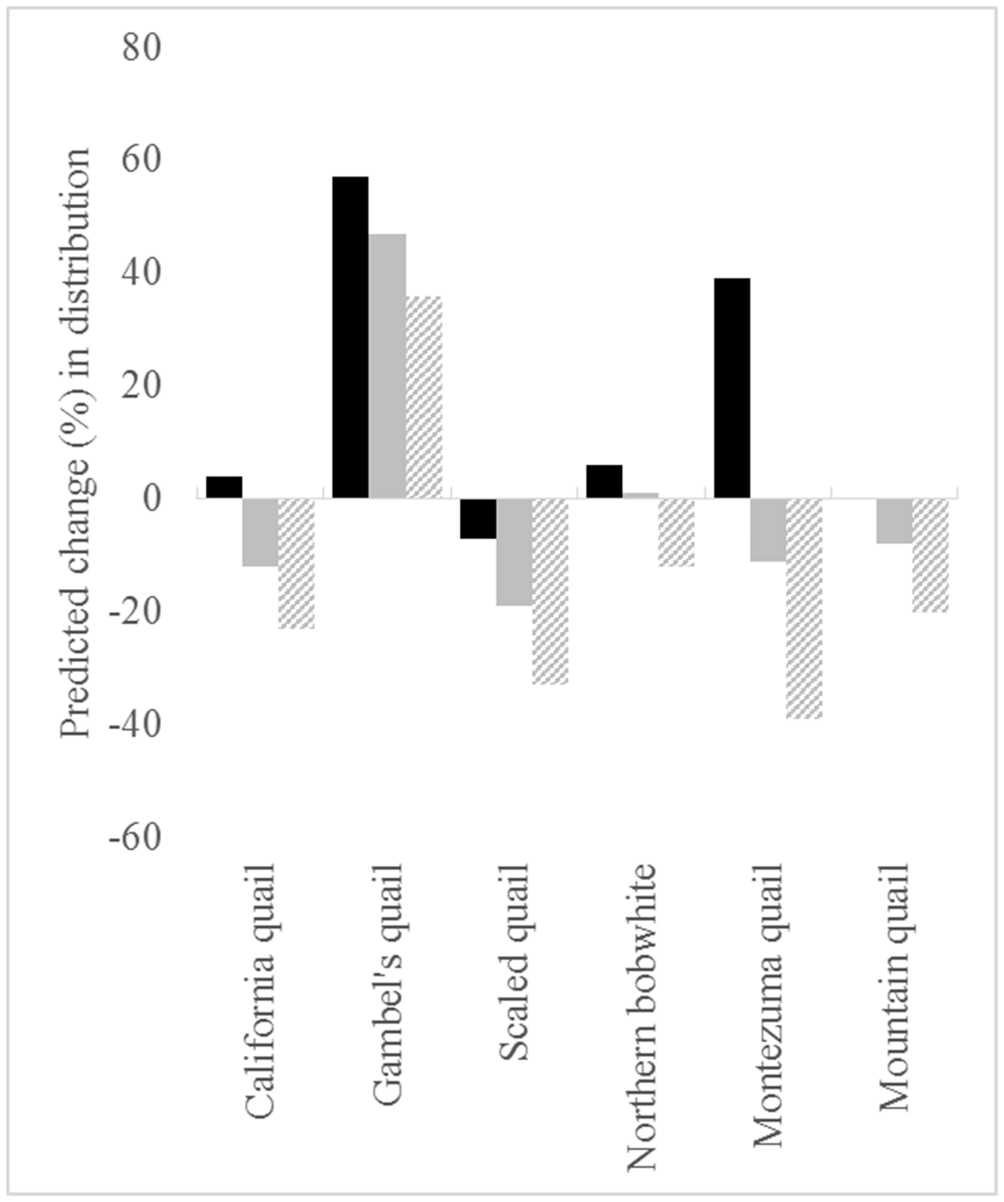
Estimated percentages of distribution shifts for temperate North American quail based on ensemble projections of Maxent models into 2070. Ensemble forecast model agreement is indicated as followed: 75% (black), 90% (gray), and 100% (striped).

**Fig 4:**
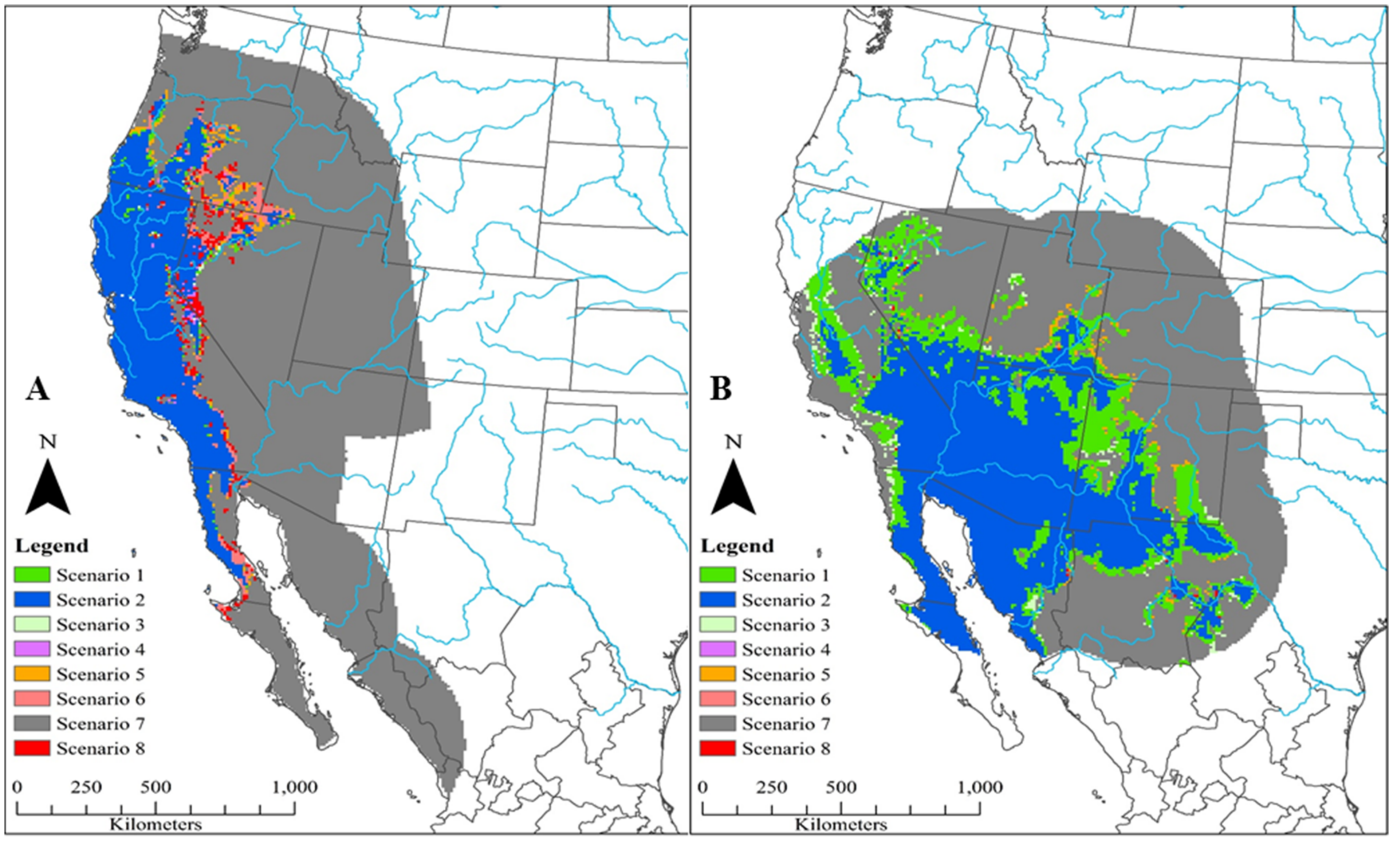
Future predicted changes in distributions of California quail (*Callipepla californica*; a) and Gambel’s quail (*Callipepla gambelii*; b) projected to 2070 and based on ensemble forecasts (estimated through Maxent) at 90% agreement. Major rivers of North America (blue lines) are included for geographic reference. Full descriptions for possible distribution conditions are given in Table 2. In short, distribution conditions represent: condition 1 (distribution expansion from current to 2050 and remaining suitable from 2050 to 2070), condition 2 (suitable at current and through all time periods), condition 3 (unsuitable from current to 2050 but expanding from 2050 to 2070), condition 4 (distribution contraction from current to 2050 but expanding from 2050 to 2070), condition 5 (distribution expansion from current to 2050 but contracting from 2050 to 2070), condition 6 (suitable from current to 2050 but contracting from 2050 to 2070), condition 7 (unsuitable at current and through all time periods), and condition 8 (distribution contraction from current to 2050 and remaining unsuitable from 2050 to 2070).

**Fig 5:**
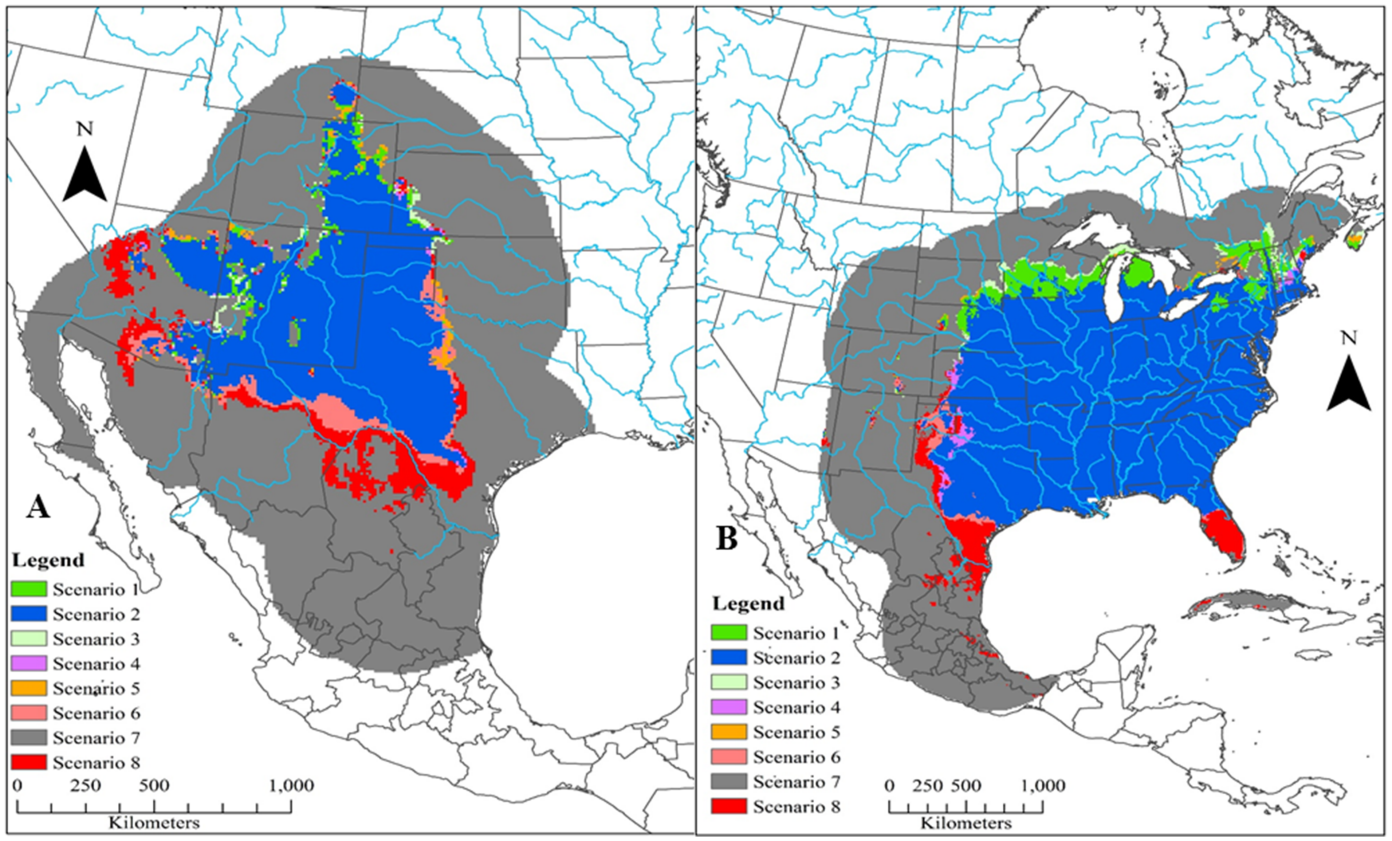
Future predicted changes in distributions of scaled quail (*Callipepla squamata*; a) and northern bobwhite (*Colinus virginianus*; b) projected to 2070 and based on ensemble forecasts (as estimated through Maxent) at 90% agreement. Major rivers of North America (blue lines) are included for geographic reference. Full descriptions for possible distribution conditions are given in Table 2. In short, distribution conditions represent: condition 1 (distribution expansion from current to 2050 and remaining suitable from 2050 to 2070), condition 2 (suitable at current and through all time periods), condition 3 (unsuitable from current to 2050 but expanding from 2050 to 2070), condition 4 (distribution contraction from current to 2050 but expanding from 2050 to 2070), condition 5 (distribution expansion from current to 2050 but contracting from 2050 to 2070), condition 6 (suitable from current to 2050 but contracting from 2050 to 2070), condition 7 (unsuitable at current and through all time periods), and condition 8 (distribution contraction from current to 2050 and remaining unsuitable from 2050 to 2070).

**Fig 6:**
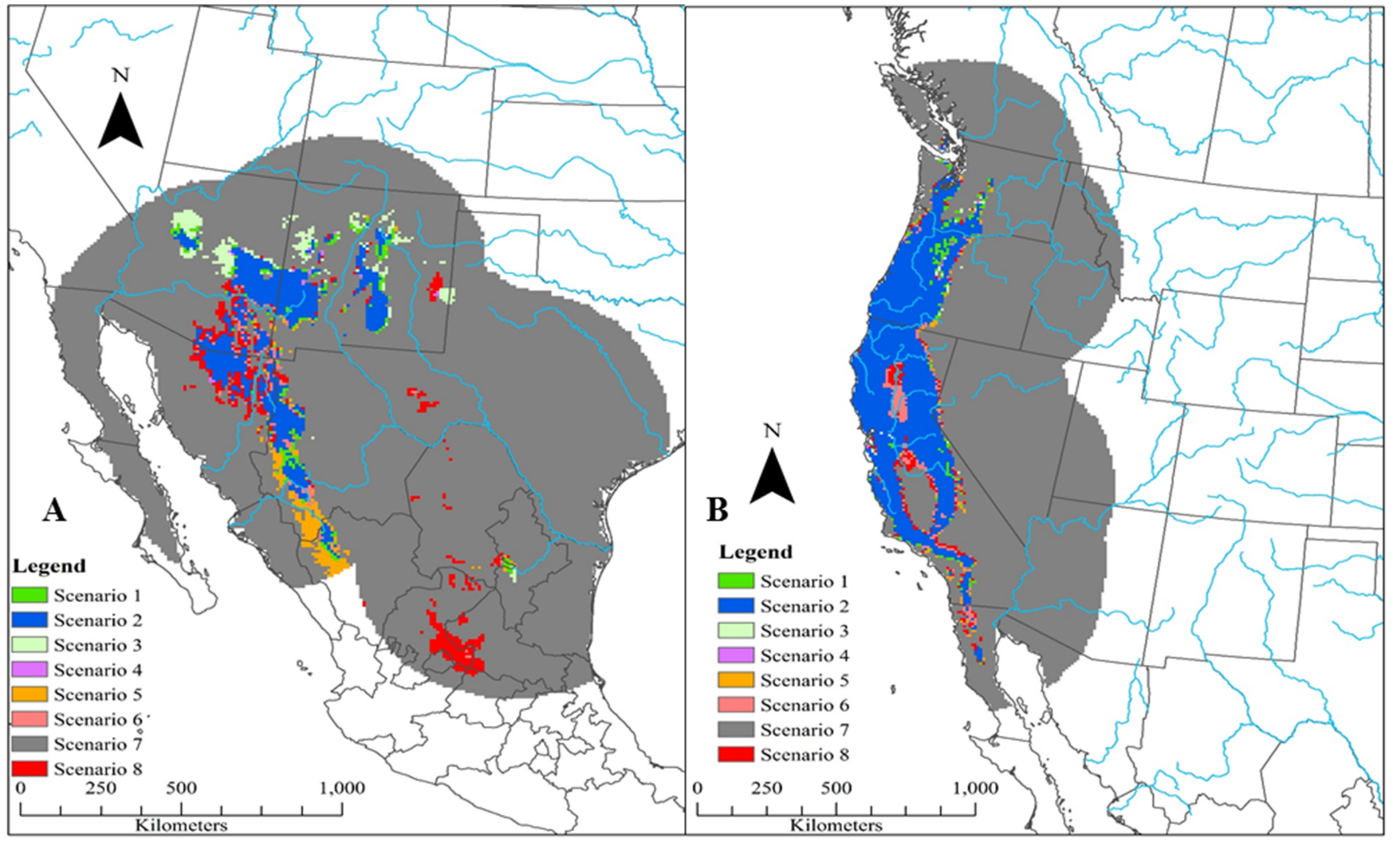
Future predicted changes in distributions of Montezuma quail (*Cyrtonyx montezumae*; a) and mountain quail (*Oreortyx pictus*; b) projected to 2070 and based on ensemble forecasts (estimated through Maxent) at 90% agreement. Major rivers of North America (blue lines) are included for geographic reference. Full descriptions for possible distribution conditions are given in Table 2. In short, distribution conditions represent: condition 1 (distribution expansion from current to 2050 and remaining suitable from 2050 to 2070), condition 2 (suitable at current and through all time periods), condition 3 (unsuitable from current to 2050 but expanding from 2050 to 2070), condition 4 (distribution contraction from current to 2050 but expanding from 2050 to 2070), condition 5 (distribution expansion from current to 2050 but contracting from 2050 to 2070), condition 6 (suitable from current to 2050 but contracting from 2050 to 2070), condition 7 (unsuitable at current and through all time periods), and condition 8 (distribution contraction from current to 2050 and remaining unsuitable from 2050 to 2070).

Based on 90% agreement between model projections on all future climate datasets into 2070, four of the six species (California quail, scaled quail, Montezuma quail, and mountain quail) are predicted to have a net loss in areas that are currently environmentally suitable (Fig 3). In general, areas of net gains in potential future distributions occurred across high latitudes whereas potential distribution contractions occurred across lower latitudes (Figs 4–6). Areas that were predicted to remain suitable for Montezuma quail under the 90% model agreement scenario were significantly higher in elevation ($\bar{x}=\;1909.16\text{ m}$, S.E. = 11.69 m) compared to areas that were predicted to become unsuitable ($\bar{x}\;=\;1652.95\text{ m}$, S.E. = 17.63 m; *t* = -12.11, *p* < 0.01). Likewise, areas that were predicted to remain suitable for mountain quail under the 90% model agreement scenario were significantly higher in elevation ($\bar{x}\;=923.07\text{ m}$, S.E. = 12.81 m) compared to areas that were predicted to become unsuitable ($\bar{x}\;=\;620.48\text{ m}$, S.E. = 28.21 m; *t* = -9.77, *p* < 0.01). Though disparity existed in estimated losses and gains of future projected distributions for all species between model agreement scenarios, Gambel’s quail was predicted to gain more environmentally suitable area in all model agreements when compared to the other five species (Fig 3). Conversely, scaled quail were predicted to lose the most area of environmental suitability (Fig 3) based on the 90% model agreement projection. It should be noted that 75% model projection agreement (S9–S11 Figs) is likely more liberal and 100% model projection agreement (S12–S14 Figs) is likely a conservative estimate of future distributions and should be interpreted with some caution.

### Implications for species relative abundance

Based on our 90% model agreement scenario, two-way ANOVA results indicated that there were significant differences in BBS relative abundance values across our predicted distribution conditions (Table 2) and across species (*F*[7,49406] = 955.45, *p* <0.01). The effect size of our model was considered medium based on an ƞ^2^ value of 0.12. All pairwise comparisons of mean relative abundance estimates across our predicted distribution conditions were significant (*p* <0.01), with mean relative abundance values of 15.96, 9.98, 7.97, and 5.51 for distribution conditions 4, 8, 6, and 2 respectively. This indicated that areas with the highest relative abundance for temperate North American quail species were predicted to contract in environmental suitability in 2050 though become suitable again in 2070 (condition 4). Furthermore, our results indicated that areas with the lowest relative abundance were in areas predicted to be the most stable in environmental suitability in future climates (condition 2).

As our initial model indicated differences across species, we conducted a one-way ANOVA across species to determine which species were predicted to lose significant areas of high relative abundance. For brevity, the results based on our 75%, 90%, and 100% model agreement scenarios are summarized in the supporting information (S1–S4 Tables). The results from our 90% model agreement scenario suggest that scaled quail are at risk of potentially losing “strongholds”, or areas of high relative abundance, under future climate scenarios. Conversely, the California quail and mountain quail are projected to lose approximately 12% and 8.1% of their potentially suitable distributions, respectively (Fig 3). Yet, for the California quail, areas that are currently estimated as suitable and are predicted to remain suitable through 2070 have significantly higher relative abundance than areas that are lost in future climate scenarios (S1 Table), while no immediate differences in relative abundance values were predicted across conditions for the mountain quail. There were no general trends with the Gambel’s quail and northern bobwhite abundance data with respect to loss of potentially suitable areas that could be considered strongholds, though they may be at risk of losing areas with intermediate levels of relative abundance in future climate scenarios. As with the ENM results, we note that the 75% model agreement scenario results are more liberal and the 100% model agreement scenario results are more conservative in relation to the 90% model agreement scenario (S1–S4 Tables).

## Discussion

Our results illustrate that climatic variables that influence local abundance do not always scale up to influence species’ distributions. This was evident as only two of the six species in our study retained abundance-based variable suites for creating ENMs. Furthermore, our ENMs predicted that only two of the six quail species are projected to have overall increases in estimated environmentally suitable area under climate scenarios into 2070, under our 90% model agreement scenario. This has important implications for conservation of these species if areas lost under future climate scenarios are currently areas with the highest relative abundance (i.e., scaled quail). By integrating estimates of future distributional shifts within the context of climate change and species’ response data such as relative abundance, managers should be able to plan for conserving novel landscapes for dispersing populations while continuing to focus efforts on areas of high abundance that are predicted to be maintained under changing climatic conditions.

The CVC variable selection approach generally outperformed the biological selection approach of climate-based variables, though the magnitude of these difference varied between species (Fig 1). The lack of performance for our biological variable suite when compared to the CVC variable suite (Fig 1) is likely related to scale [103], suggesting that factors that influence local species’ abundance do not always scale up to determine species’ distributions. For example, the northern bobwhite has a broad distribution which will experience varying effects of climate change across latitudinal (i.e., temperature) and longitudinal (i.e., precipitation) gradients. Furthermore, for the two species in which the biological variable suite was used to determine species’ distributions (Gambel’s quail and Montezuma quail), there was evidence of transmutability [104] in the relationship of these variables as data were scaled up. For instance, a negative relationship between summer temperatures and productivity of Gambel’s quail has been reported [50]. However our results indicate that, although maximum average summer temperature contributed most to our ENMs for Gambel’s quail (Table 3), there was actually a positive relationship between probability of suitability and maximum average summer temperature (S4 Fig). Transmutation across scales also occurred for the Montezuma quail data, in which the positive relationship between abundance and summer precipitation [60] changed to a unimodal relationship (i.e., an indication of niche breadth) when scaled up to the species’ distribution (S7 Fig). These results further emphasize the importance of considering scale when working with species’ distribution models.

Based on our analysis of species’ relative abundance, scaled quail and northern bobwhite trends indicated the potential loss of areas with high and intermediate relative abundance, respectively (S1 Table). A majority of these areas occurred on the periphery of the estimated species’ distributions [41]. As climate induced shifts in distributions can often affect edge populations disproportionately [105], direct loss of these “peripheral strongholds” could have major conservation implications [106]. We note that just as distributions are expected to shift, dispersal patterns and species’ interactions with biotic and abiotic variables [107] will likely facilitate a shift in species’ abundance as well. Interpretations should take this into consideration and future research attempting to model shifts in the future abundance of these species would be beneficial.

A general outcome in biogeographical studies in the context of future climate change is that non-montane species tend to shift distributions northward while montane species shift distributions towards higher elevations [9,108–111]. Species endemic to high elevation areas may be more vulnerable to a changing climate as they become more restricted to smaller, higher elevation areas termed “sky islands” [112]. Geographic restriction of species to these sky islands may be a result of the traditional low elevation/competition vs. high elevation/physiological stress hypothesis [113], though more recently this pattern has also been attributed to the phylogenetic niche conservatism process [114–115], in which instantaneous niche retention exists [116]. If indeed niche conservatism determines high elevation distribution restrictions in certain species, they may be highly susceptible to geographic isolation due to climate change [115]. In our study, both Montezuma quail and mountain quail, which typically occur at elevations >1,000 m [35, 100], had predictions of distribution contraction based on 90% and 100% model agreement scenarios. Consistent with the theory of sky islands, the contraction of these species’ distributions occurred at lower elevations and southern latitudes and areas of suitability were retained at significantly higher elevations. Whether this is related to phylogenetic niche conservatism or an interaction between low elevation and competition is unknown. However, for mountain quail, interspecific competition with similar species like the California quail seems to be insignificant [117] and interspecific competition between Montezuma quail and other sympatric quail species is largely understudied. Furthermore, a unique biogeographic history with early genetic separation for the Montezuma quail (~15 Ma; [118]) and mountain quail (~12.6 Ma; [118–119]) may lend such high elevation restrictions toward niche conservatism.

All six species indicated general trends of southern latitudinal loss, at varying levels, in estimated environmental suitability of their current distribution (Figs 4–6). This has been shown in many other Galliformes, in which northward shifts were more common than any other directional shift [42]. However, these southern edge shifts in future predicted distributions should be viewed with caution. The low latitudinal periphery of a species’ distribution could actually have high stability because of heterogeneity in topography and in plant community structure, providing greater opportunities for establishing climatic niches [105,120]. The variability in these responses is related to the scale at which most climate change research is focused. Detailed knowledge is becoming increasingly available on how organisms respond to fine-scale heterogeneity in a thermal landscape, particularly in relation to local topography and vegetation structure [121–125]. These behavioral responses could help to stabilize potential distribution shifts. For instance, in our study a temperature-related variable was the highest contributing variable for only half of the species (Table 3), with all relationships indicating the presence of a niche breadth except for the Gambel’s quail (S3–S8 Figs). It is likely that temperature was not the best contributor to many broad scale models in our study because many of these species have been known to phenotypically and behaviorally adapt to variation in temperatures at very fine scales [126–129], which may slow the rate of low latitudinal distribution contraction beyond that which our models predict.

The use of land cover data in ENMs has produced mixed results with regards to model performance and predicted distributions for many species, and varies across species [130–133]. However, there is often high uncertainty in projected future land cover models and these variables are often not included when projecting ENMs into future scenarios [134]. Thus, our overall goal was to model the climatic suitability for these species rather than incorporating both climate variables and land cover data under future scenarios. This is not to say that land cover can be ignored in conservation planning and management efforts. Indeed, current and future land cover across species’ distributions will certainly influence abundance and distribution of Galliformes included in our analysis [135–137]. Climate based models merely offer one of several tools to aid in decision making and conservation prioritization and should be viewed as such, with the inherent limitations acknowledged.

Furthermore, we note that we were restricted to analyzing our datasets within a presence-only/pseudo-absence framework [84], which offers unique challenges to predicting species’ distributions due to assumptions based on sampling biases and detection probabilities of individuals [138]. However, as demonstrated with this study, researchers are often limited to using presence-only data to ensure coverage of presence points throughout the known distribution by collating multiple datasets. Because of this, techniques have been developed to overcome these biases [68, 139] and research has demonstrated robust model performance from the Maxent algorithm despite these biases [140–141]. Yet, we do acknowledge that variability in detection probabilities could possibly lead to model uncertainty, particularly within areas that are predicted to be areas of range expansion/contraction [142]. Thus, areas categorized under distribution conditions 3, 4, 5, and 6 (Table 2) should be viewed with this bias in mind.

Though conservation has historically been considered a crisis discipline with objectives focused on preventing the extinction of rare or threatened species [143–144], recent arguments suggest conservation biologists should also focus efforts on conservation of more common species, as declines in such species may be representative of changes in ecological structure and functions [144–145]. A benefit to modeling common species is that occurrence data and knowledge of biologically meaningful climatic variables can often be easily accessible, as we have demonstrated here. These data may give conservation biologists insight into broad temporal and spatial trends related to at risk ecosystems. We suggest, as did [44, 144], that relatively common species, in addition to rare species, should receive attention if maintaining biodiversity is a goal.

## Supporting information

S1 Fig Spatially rarefied occurrence locations used in creating ecological niche models for temperate North American quail in the Maxent algorithm. (PDF)

S2 Fig Spatial relative abundance data used in post-hoc analysis of ensemble forecast species distribution models for temperate North American quail. (PDF)

S3 Fig Relationship between environmental variables and probability of climate suitability for California quail (*Callipepla californica*). Response curves indicate mean response of 100 replicated Maxent runs and the +/- one standard deviation (grey).
(PDF)

S4 Fig Relationship between environmental variables and probability of climate suitability for Gambel’s quail (*Callipepla gambelii*). Response curves indicate mean response of 100 replicated Maxent runs and the +/- one standard deviation (grey).
(PDF)

S5 Fig Relationship between environmental variables and probability of climate suitability for scaled quail (*Callipepla squamata*). Response curves indicate mean response of 100 replicated Maxent runs and the +/- one standard deviation (grey).
(PDF)

S6 Fig Relationship between environmental variables and probability of climate suitability for northern bobwhite (*Colinus virginianus*). Response curves indicate mean response of 100 replicated Maxent runs and the +/- one standard deviation (grey).
(PDF)

S7 Fig Relationship between environmental variables and probability of climate suitability for Montezuma quail (*Cyrtonyx montezumae*). Response curves indicate mean response of 100 replicated Maxent runs and the +/- one standard deviation (grey).
(PDF)

S8 Fig Relationship between environmental variables and probability of climate suitability for mountain quail (*Oreortyx pictus*). Response curves indicate mean response of 100 replicated Maxent runs and the +/- one standard deviation (grey).
(PDF)

S9 Fig Future predicted changes^1^ in distributions of California quail (*Callipepla californica*; A) and Gambel’s quail (*Callipepla gambelii*; B) projected to 2070 and based on ensemble ecological niche models at 75% model agreement as estimated through Maxent. (PDF)

S10 Fig Future predicted changes^1^ in distributions of scaled quail (*Callipepla squamata*; A) and northern bobwhite (*Colinus virginianus*; B) projected to 2070 and based on ensemble ecological niche models at 75% model agreement as estimated through Maxent. (PDF)

S11 Fig Future predicted changes^1^ in distributions of Montezuma quail (*Cyrtonyx montezumae*; A) and mountain quail (*Oreortyx pictus*; B) projected to 2070 and based on ensemble ecological niche models at 75% model agreement as estimated through Maxent. (PDF)

S12 Fig Future predicted changes^1^ in distributions of California quail (*Callipepla californica*; A) and Gambel’s quail (*Callipepla gambelii*; B) projected to 2070 and based on ensemble ecological niche models at 100% model agreement as estimated through Maxent. (PDF)

S13 Fig Future predicted changes^1^ in distributions of scaled quail (*Callipepla squamata*; A) and northern bobwhite (*Colinus virginianus*; B) projected to 2070 and based on ensemble ecological niche models at 100% model agreement as estimated through Maxent. (PDF)

S14 Fig Future predicted changes^1^ in distributions of Montezuma quail (*Cyrtonyx montezumae*; A) and mountain quail (*Oreortyx pictus*; B) projected to 2070 and based on ensemble ecological niche models at 100% model agreement as estimated through Maxent. (PDF)

S1 Table Mean relative abundance (RA) estimates and standard errors (SE) of temperate North American quail species and associated conditions of distributions, based on ecological niche models using the Maxent algorithm, at 90% ensemble forecasting agreement. Significant difference in RA estimates indicated by post hoc Tukey HSD pairwise comparison test results from a one-way ANOVA.
(PDF)

S2 Table Mean relative abundance (RA) estimates and standard errors (SE) of temperate North American quail species and associated conditions of distributions, based on ecological niche models using the Maxent algorithm, at 75% ensemble forecasting agreement. Significant difference in RA estimates indicated by post hoc Tukey HSD pairwise comparison test results from a one-way ANOVA.
(PDF)

S3 Table Mean relative abundance (RA) estimates and standard errors (SE) of temperate North American quail species and associated conditions of distributions, based on ecological niche models using the Maxent algorithm, at 100% ensemble forecasting agreement. Significant difference in RA estimates indicated by post hoc Tukey HSD pairwise comparison test results from a one-way ANOVA.
(PDF)

S4 Table One-way ANOVA test statistics and effect sizes (ƞ^2^) for comparison of mean relative abundance estimates for temperate North American quail across potential distribution conditions estimated from ensemble forecasted ecological niche models. Model agreement scenarios indicate the percentage of models (out of 32) that agree on future climatic suitability for species.
(PDF)
