# Supplementary material for: Incorporating abundance information and guiding variable selection for climate-based ensemble forecasting of species' distributional shifts
Source: PLoS One. 2017 Sep 8;12(9):e0184316. doi: 10.1371/journal.pone.0184316 (PMC5590900; doi:10.1371/journal.pone.0184316)
Supplement: S1 Fig — (PDF) [file pone.0184316.s001.pdf]

Fig. S1 Spatially rarefied occurrence locations for (A) California quail (*Callipepla californica*), (B) Gambel's quail (*Callipepla gambelii*), (C) scaled quail (*Callipepla squamata*), (D) northern bobwhite (*Colinus virginianus*), (E) Montezuma quail (*Curtonyx montezumae*), and (F) mountain quail (*Oreotyx pictus*) used in creating ecological niche models in the Maxent algorithm. Data were obtained from the Breeding Bird Survey and eBird databases and range from 1950-2000. Spatial rarefication was set at 20 km. Major rivers of North America (blue lines) are included for geographic reference.

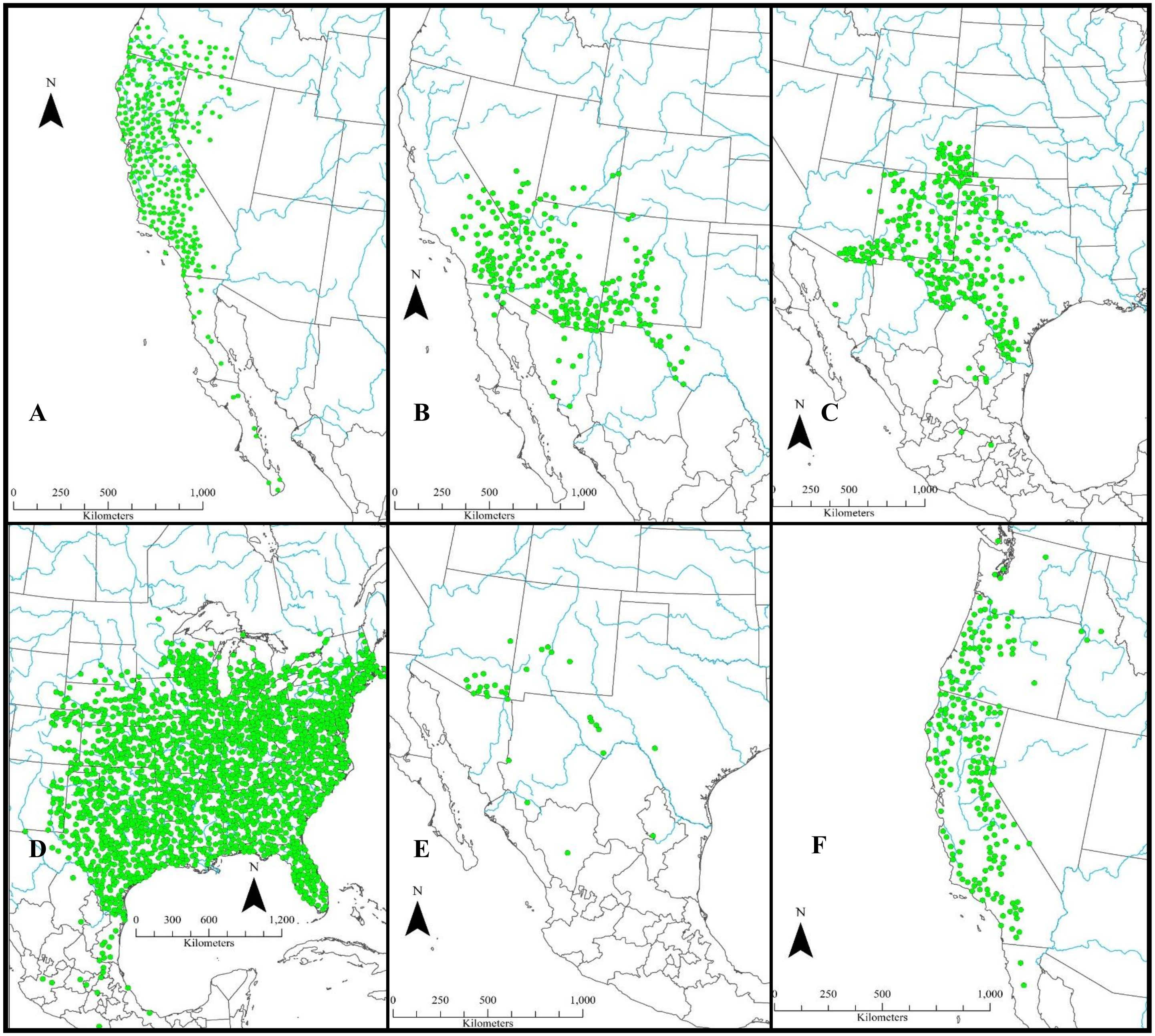

A
0 250 500 1,000
Kilometers
N
B
0 250 500 1,000
Kilometers
N
C
0 250 500 1,000
Kilometers
N
D
0 300 600 1,200
Kilometers
N
E
0 250 500 1,000
Kilometers
N
F
0 250 500 1,000
Kilometers
N
