# Supplementary material for: Incorporating abundance information and guiding variable selection for climate-based ensemble forecasting of species' distributional shifts
Source: PLoS One. 2017 Sep 8;12(9):e0184316. doi: 10.1371/journal.pone.0184316 (PMC5590900; doi:10.1371/journal.pone.0184316)
Supplement: S2 Fig — (PDF) [file pone.0184316.s002.pdf]

Fig S2. Relative abundance data[abc] used in post-hoc analysis of ensemble forecast species distribution models for (A) California quail (*Callipepla californica*), (B) Gambel's quail (*Callipepla gambelii*), (C) scaled quail (*Callipepla squamata*), (D) northern bobwhite (*Colinus virginianus*), and (E) mountain quail (*Oreotyx pictus*). Relative abundance data were from the Breeding Bird Survey from 2008-2012. Major rivers of North America (blue lines) are included for geographic reference.

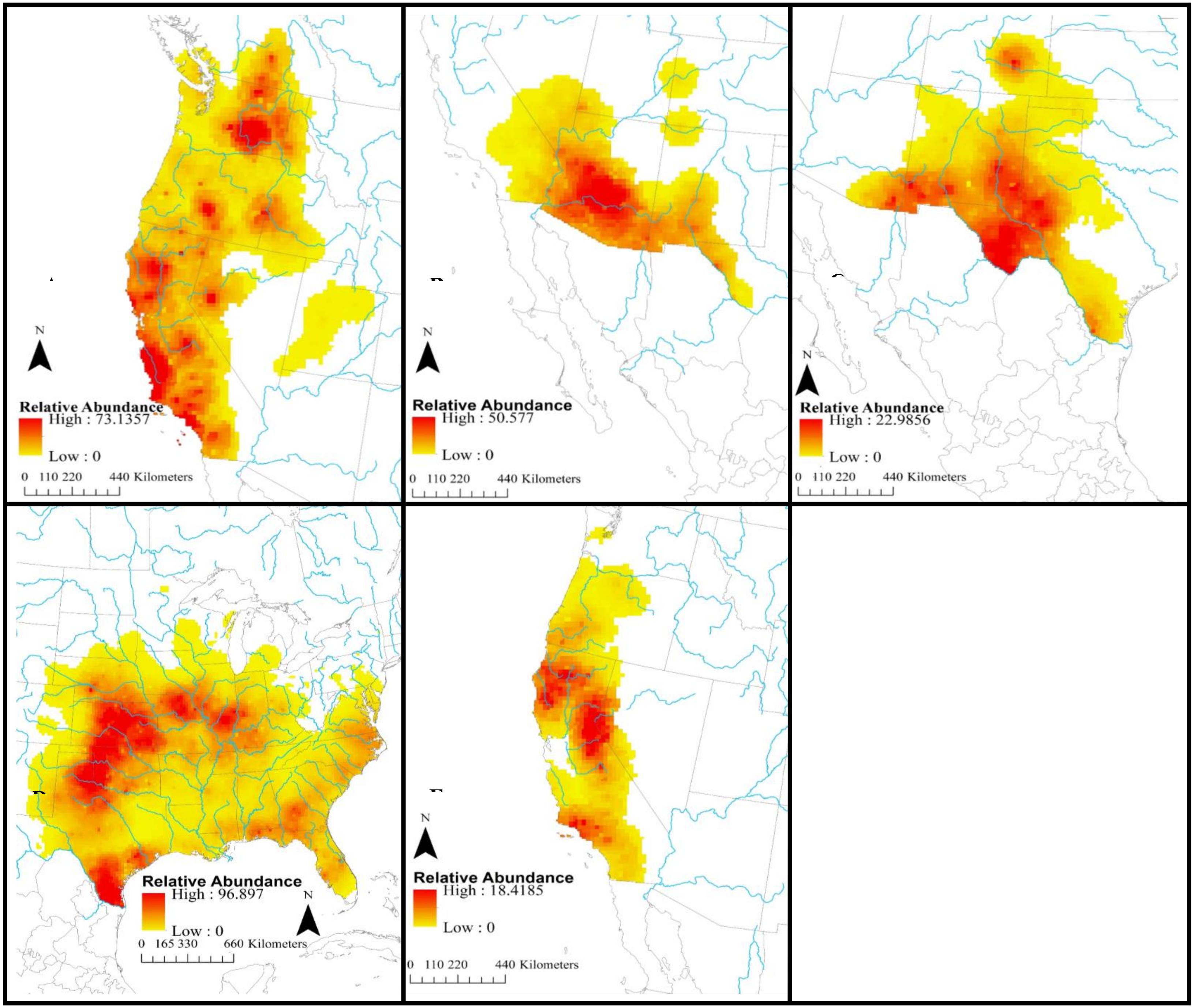
Relative Abundance
High : 73.1357
Low : 0
0 110 220 440 Kilometers
N
Relative Abundance
High : 50.577
Low : 0
0 110 220 440 Kilometers
N
Relative Abundance
High : 22.9856
Low : 0
0 110 220 440 Kilometers
N
Relative Abundance
High : 96.897
Low : 0
0 165 330 660 Kilometers
N
Relative Abundance
High : 18.4185
Low : 0
0 110 220 440 Kilometers
N

[a] Estimated from [41]. Values generally predict the average number of birds for a species that can be seen along roadsides in ~2.5 hours.

[b] Data not available for Montezuma quail.

[c] Data outside the United States not available and data from introduced populations excluded in our analysis.
