# Supplementary material for: Incorporating abundance information and guiding variable selection for climate-based ensemble forecasting of species' distributional shifts
Source: PLoS One. 2017 Sep 8;12(9):e0184316. doi: 10.1371/journal.pone.0184316 (PMC5590900; doi:10.1371/journal.pone.0184316)

Fig. S5 Relationship between environmental variables and probability of climate suitability for scaled quail (*Callipepla squamata*).

Response curves indicate mean response of 100 replicated Maxent runs and the +/- one standard deviation (grey).

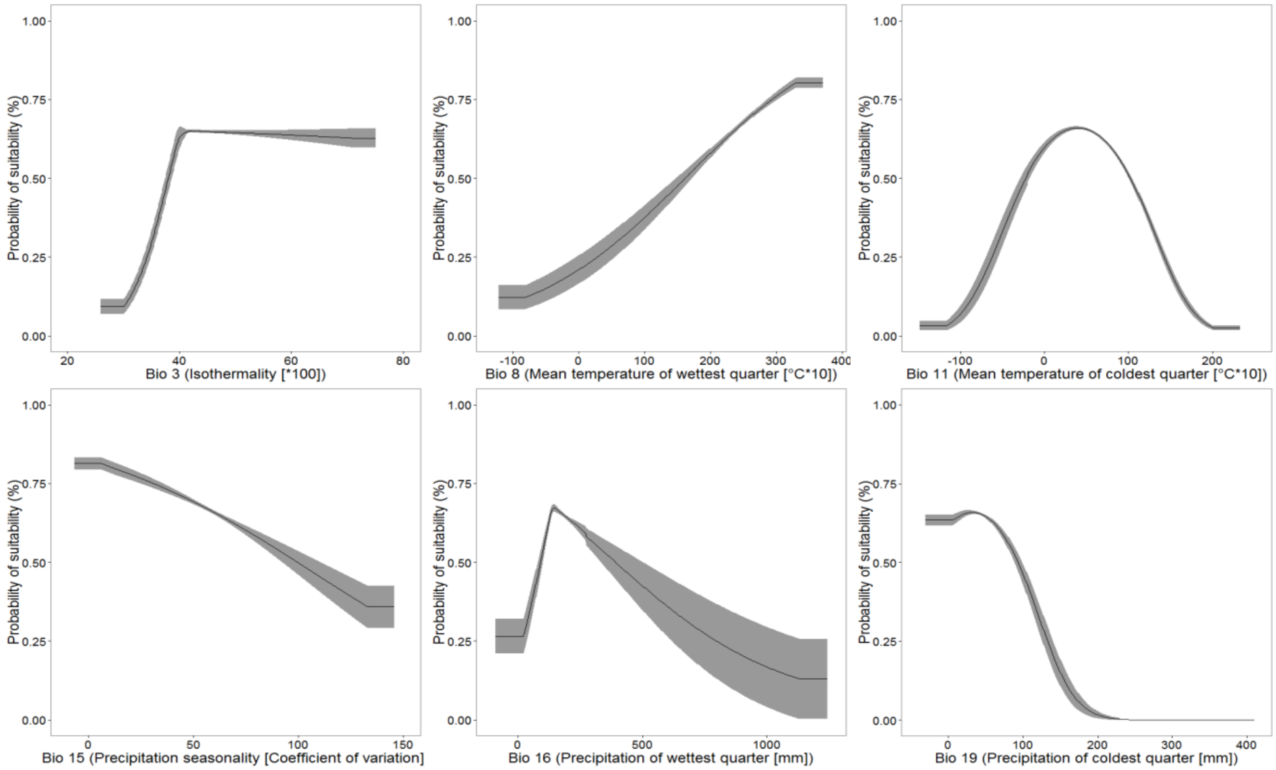

Supplement: S5 Fig — Response curves indicate mean response of 100 replicated Maxent runs and the +/- one standard deviation (grey). (PDF) [file pone.0184316.s005.pdf]
