# Supplementary material for: Incorporating abundance information and guiding variable selection for climate-based ensemble forecasting of species' distributional shifts
Source: PLoS One. 2017 Sep 8;12(9):e0184316. doi: 10.1371/journal.pone.0184316 (PMC5590900; doi:10.1371/journal.pone.0184316)
Supplement: S13 Fig — (PDF) [file pone.0184316.s013.pdf]

Fig. S13 Future predicted changes[a] in distributions of scaled quail (*Callipepla squamata*; A) and northern bobwhite (*Colinus virginianus*; B) projected to 2070 and based on ensemble ecological niche models at 100% model agreement as estimated through Maxent. Major rivers of North America (blue lines) are included for geographic reference.

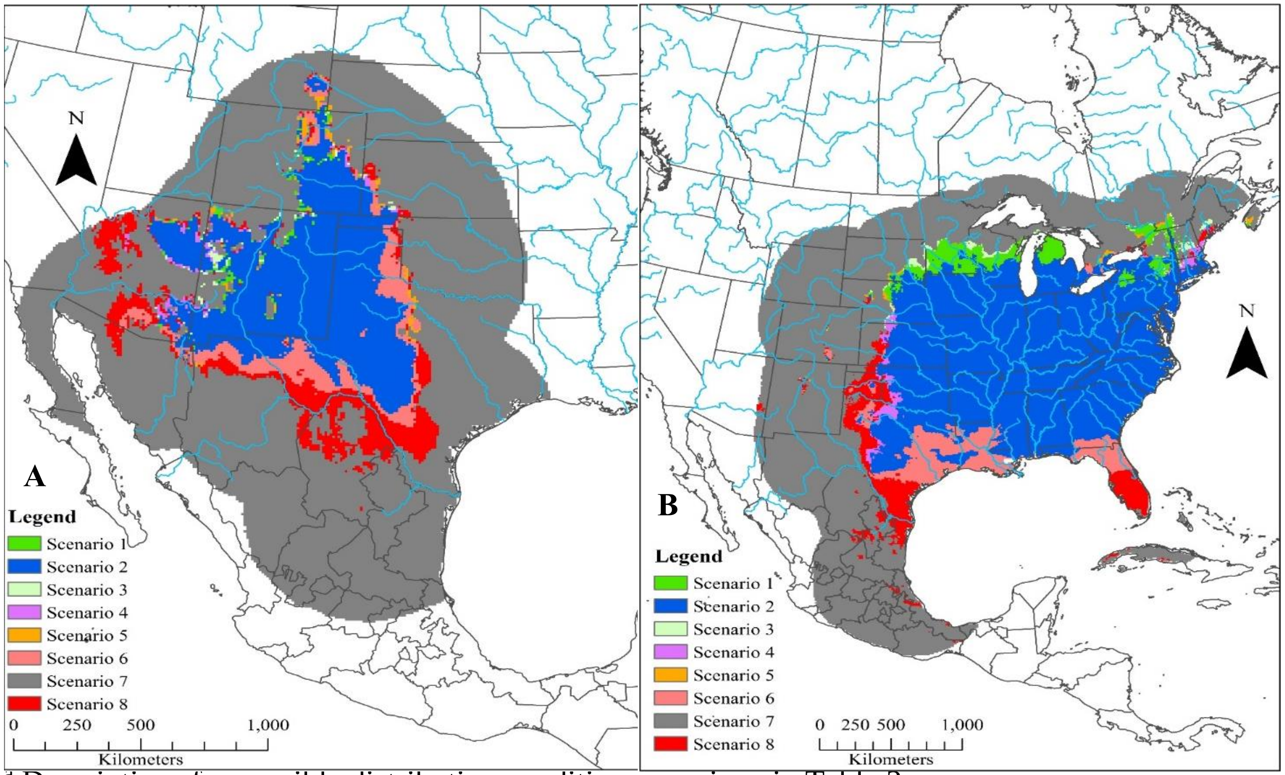

[a] Descriptions for possible distribution conditions are given in Table 2.
