# Supplementary material for: Incorporating abundance information and guiding variable selection for climate-based ensemble forecasting of species' distributional shifts
Source: PLoS One. 2017 Sep 8;12(9):e0184316. doi: 10.1371/journal.pone.0184316 (PMC5590900; doi:10.1371/journal.pone.0184316)
Supplement: S1 Table — Significant difference in RA estimates indicated by post hoc Tukey HSD pairwise comparison test results from a one-way ANOVA. (PDF) [file pone.0184316.s015.pdf]

Table S1. Mean relative abundance (RA) estimates[a] and standard errors (SE) of temperate North American quail species[b] and associated conditions[c] of distributions, based on ecological niche models using the Maxent algorithm, at 90% ensemble forecasting agreement. Significant difference in RA estimates indicated by post hoc Tukey HSD pairwise comparison test results[d] from a one-way ANOVA.

Species	Condition	Mean RA	SE	Tukey-Kramer grouping
California quail	2	7.23	0.08	A
	4	4.51	0.55	B
	6	3.59	0.22	B
	8	4.27	0.19	B
Gambel's quail	2	10.07	0.14	A
	4	3.80	2.09	B
	6	5.20	1.12	BA
	8	1.38	0.48	B
Scaled quail	2	2.78	0.03	B
	4	1.15	0.26	C
	6	4.58	0.20	A
	8	2.80	0.16	B
Northern bobwhite	2	6.99	0.05	C
	4	21.47	0.74	A
	6	14.19	0.40	B
	8	14.63	0.29	B
Mountain quail	2	2.25	0.04	A
	4	2.09	1.75	A
	6	1.89	0.09	A
	8	1.87	0.11	A

[a] Estimated from [41]. Values generally predict the average number of birds for a species that can be seen along roadsides in ~2.5 hours.

[b] Data not available for Montezuma quail.

[c] Descriptions for possible distribution conditions are given in Table 2.

[d] Letter categories represent significant differences between relative abundance values between conditions at $\alpha = 0.05$ level.
