# Supplementary material for: Incorporating abundance information and guiding variable selection for climate-based ensemble forecasting of species' distributional shifts
Source: PLoS One. 2017 Sep 8;12(9):e0184316. doi: 10.1371/journal.pone.0184316 (PMC5590900; doi:10.1371/journal.pone.0184316)
Supplement: S2 Table — Significant difference in RA estimates indicated by post hoc Tukey HSD pairwise comparison test results from a one-way ANOVA. (PDF) [file pone.0184316.s016.pdf]

Table S2. Mean relative abundance (RA) estimates[a] and standard errors (SE) of temperate North American quail species[b] and associated conditions[c] of distributions, based on ENMs using the Maxent algorithm, at 75% ensemble forecasting agreement. Significant difference in RA estimates indicated by post hoc Tukey HSD pairwise comparison test results[d] from a one-way ANOVA.

Species	Condition	Mean RA	SE	Tukey-Kramer grouping
California quail	2	6.89	0.08	A
	4	5.21	0.82	BA
	6	4.64	0.45	BA
	8	4.06	0.24	B
Gambel's quail	2	10.09	0.14	A
	4	5.28	2.05	BA
	6	0.37	0.06	B
	8	1.72	0.71	B
Scaled quail	2	2.85	0.03	B
	4	0.34	0.10	C
	6	4.84	0.30	A
	8	2.42	0.18	B
Northern bobwhite	2	7.41	0.05	D
	4	16.40	1.76	A
	6	12.17	0.39	C
	8	14.34	0.35	B
Mountain quail	2	2.21	0.04	A
	4	NA[e]	NA[e]	NA[e]
	6	2.33	0.12	A
	8	1.75	0.17	A

[a] Estimated from Sauer et al. (2014). Values generally predict the average number of birds for a species that can be seen along roadsides in ~2.5 hours.

[b] Data not available for Montezuma quail.

[c] Descriptions for possible distribution conditions are given in Table 2.

[d] Letter categories represent significant differences between relative abundance values between conditions at $\alpha = 0.05$ level.

[e] Not applicable. Distributional condition not present in this model agreement scenario.
