# Supplementary material for: Incorporating abundance information and guiding variable selection for climate-based ensemble forecasting of species' distributional shifts
Source: PLoS One. 2017 Sep 8;12(9):e0184316. doi: 10.1371/journal.pone.0184316 (PMC5590900; doi:10.1371/journal.pone.0184316)
Supplement: S3 Table — Significant difference in RA estimates indicated by post hoc Tukey HSD pairwise comparison test results from a one-way ANOVA. (PDF) [file pone.0184316.s017.pdf]

Table S3. Mean relative abundance (RA) estimates[a] and standard errors (SE) of temperate North American quail species[b] and associated conditions[c] of distributions, based on ENMs using the Maxent algorithm, at 100% ensemble forecasting agreement. Significant difference in RA estimates indicated by post hoc Tukey HSD pairwise comparison test results[d] from a one-way ANOVA.

Species	Condition	Mean RA	SE	Tukey-Kramer grouping
California quail	2	7.45	0.09	A
	4	5.64	0.51	B
	6	4.07	0.19	C
	8	4.47	0.17	C
Gambel's quail	2	10.07	0.14	A
	4	4.43	1.53	A
	6	NA[e]	NA[e]	NA[e]
	8	2.10	1.35	A
Scaled quail	2	2.82	0.03	A
	4	1.73	0.19	B
	6	3.34	0.12	A
	8	3.10	0.14	A
Northern bobwhite	2	6.94	0.05	C
	4	19.14	0.39	A
	6	5.82	0.11	D
	8	15.60	0.25	B
Mountain quail	2	2.28	0.04	A
	4	0.48	0.14	A
	6	2.03	0.08	A
	8	1.82	0.09	A

[a] Estimated from Sauer et al. (2014). Values generally predict the average number of birds for a species that can be seen along roadsides in ~2.5 hours.

[b] Data not available for Montezuma quail.

[c] Descriptions for possible distribution conditions are given in Table 2.

[d] Letter categories represent significant differences between relative abundance values between conditions at $\alpha = 0.05$ level.

[e] Not applicable. Distributional condition not present in this model agreement scenario.
