# Supplementary material for: Incorporating abundance information and guiding variable selection for climate-based ensemble forecasting of species' distributional shifts
Source: PLoS One. 2017 Sep 8;12(9):e0184316. doi: 10.1371/journal.pone.0184316 (PMC5590900; doi:10.1371/journal.pone.0184316)
Supplement: S4 Table — Model agreement scenarios indicate the percentage of models (out of 32) that agree on future climatic suitability for species. (PDF) [file pone.0184316.s018.pdf]

Table S4. One-way ANOVA test statistics and effect sizes (η^2) for comparison of mean relative abundance estimates for temperate North American quail across potential distribution conditions[a] estimated from ensemble forecasted ecological niche models. Model agreement scenarios indicate the percentage of models (out of 32) that agree on future climatic suitability for species.

Model agreement scenario	Species	*F*	d.f. between	d.f. within	*p*	η^2
75%	California quail	19.21	3	4324	<0.01	0.01
	Gambel's quail	9.45	3	4417	<0.01	0.01
	Scaled quail	44.1	3	6967	<0.01	0.02
	Northern bobwhite	324.94	3	30467	<0.01	0.03
	Mountain quail	1.82	2	3219	0.16	<0.01
90%	California quail	77.1	3	4324	<0.01	0.05
	Gambel's quail	21.13	3	4417	0.1	0.01
	Scaled quail	68.45	3	6967	<0.01	0.03
	Northern bobwhite	99.94	3	30467	<0.01	0.09
	Mountain quail	3.5	3	3219	0.02	<0.01
100%	California quail	102.02	3	4324	<0.01	0.07
	Gambel's quail	2.3	2	4417	0.1	<0.01
	Scaled quail	15.57	3	6967	<0.01	<0.01
	Northern bobwhite	1310.48	3	30467	<0.01	0.11
	Mountain quail	6.58	3	3219	<0.01	0.01

[a] Descriptions for possible distribution conditions are given in Table 2.
